# The decisive role of molecular pathology in presumed somatic metastases of type II testicular germ cell tumors: report of 2 cases

**DOI:** 10.1186/s13000-020-01011-0

**Published:** 2020-07-25

**Authors:** Mariëtte E. G. Kranendonk, Wenzel M. Hackeng, G. Johan A. Offerhaus, Folkert H. M. Morsink, Geertruida N. Jonges, Gerard Groenewegen, Pieter-Jaap Krijtenburg, Heinz-Josef Klümpen, Wendy W. J. de Leng, Leendert H. J. Looijenga, Lodewijk A. A. Brosens

**Affiliations:** 1 Department of Pathology, University Medical Center Utrecht, Utrecht, The Netherlands; 2 Princess Máxima Center for Pedriatric Oncology, Utrecht, The Netherlands; 3 Department of Medical Oncology, University Medical Center Utrecht, Utrecht, The Netherlands; 4 Department of Medical Genetics, University Medical Center Utrecht, Utrecht, The Netherlands; 5 Department of Medical Oncology, Cancer Center Amsterdam, Amsterdam UMC, University of Amsterdam, Amsterdam, the Netherlands

**Keywords:** Testicular germ cell tumor, Metastasis, Molecular diagnostics, Gain 12p, Case report

## Abstract

**Background:**

Molecular diagnostics can be decisive in the differential diagnosis between a somatic metastasis of type II testicular germ cell tumor (TGCT) or a second primary carcinoma. This is in line with recent recommendations from the International Society of Urological Pathology, based on an international survey which showed that molecular testing is currently only performed by a minority of urological pathologists.

**Case presentations:**

This case report illustrates the necessity of molecular testing in two patients with a history of type II TGCT and a metastatic (retro) peritoneal carcinoma years later. The genetic hallmark of type II TGCT, chromosome 12p gain, was studied by fluorescence in situ hybridization and whole genome methylation profiling in case 1, and by single nucleotide polymorphism (SNP)-array in case 2. Next generation sequencing (NGS) was used to further explore clonality between the primary TGCT and peritoneal metastasis in case 2. In case 1, chromosome 12p gain was found in the primary type II TGCT and in the acinar cell carcinoma of the metastatic malignancy. In case 2, SNP array showed 12p gain in the epithelial component of the primary teratomatous TGCT but not in the peritoneal adenocarcinoma. Furthermore, NGS showed no mutations in the primary teratomatous TGCT but a KRAS and GNAS mutation in the peritoneal adenocarcinoma, suggestive of an appendicular origin.

**Conclusions:**

Without the molecular data, both cases would have been regarded as a metastatic TGCT with development of somatic-type malignancy, which appeared a wrong diagnosis for case 2. These cases demonstrate the importance of molecular methods as an adjunct in today’s pathology practice.

## Background

Testicular germ cell tumors (TGCTs) account for less than 1% of all cancers in the male population, but are the most common malignancy in young men [1]. A teratoma with somatic-type malignancy developing in metastatic sites is a rare but well known phenomenon [2–5] and needs a different treatment than a metastasis from a primary epithelial carcinoma [6].

The genetic hallmark of type II TGCTs, including teratomas with somatic-type malignancy, is a gain of the short arm of chromosome 12 (chromosome 12p), mainly as isochromosome 12p [6], resulting in the amplification of a cluster of genes that promote sertoli cell independent cell growth [7]. Confirmation of a gain of 12p copy numbers in metastatic somatic-type malignancies in patients with a history of type II TGCT can therefore make germ cell origin more likely [8].

Interestingly, 12p gain has been described in other malignancies, including malignant teratoma of the thyroid (considered as an extragonadal germ cell tumor) [9] but also in pancreatic carcinomas [10]. However, the amplified region in pancreatic carcinomas is located proximal to the amplified region in TGCT [10]. Therefore, in the right context, 12p gain or a 12p isochromosome variant is diagnostic for TGCT [11].

Here we describe two patients with a history of teratomatous type II TGCT and a (retro) peritoneal metastatic somatic malignancy years later (6 and 33 years respectively). As both metastases presented more than 2 years after the completion of first-line chemotherapy for advanced TGCT, the differential diagnosis included late recurrent metastasized TGCT with somatic-type malignancy [12–14]. The first case encompassed a metastatic retroperitoneal teratoma with a pancreatic acinar cell carcinoma (ACC) component. As this is one of the more rare described somatic malignancies within a teratoma it needed to be distinguished from a metastasis of a primary pancreatic carcinoma [15]. The second case describes a patient with peritoneal metastatic adenocarcinoma with morphological and immunohistochemical similarities to an epithelial component of the primary teratomatous TGCT. To further underpin the type II TGCT origin of both epithelial malignancies, molecular assays were used which revealed surprising and crucial information for the final diagnosis. These cases illustrate the decisive role for molecular data in nowadays pathological assessment of (metastatic) type II TGCT, in line with recent recommendations from the International Society of Urological Pathology (ISUP) [8].

## Presentation of cases

### Case 1

During a follow up CT scan, 6 years after a right orchidectomy and multiple courses of systemic therapy for a testis tumor, a retroperitoneal mass was found in a male patient in his twenties. The primary testis tumor was a type II TGCT, consisting of combined teratoma and seminoma of 1.4 cm comprising a stage IIIc intermediate risk non-seminoma of the testis. At that time, serum alpha-fetoprotein (AFP), human chorionic gonadotropin-beta (beta-HCG) and lactate dehydrogenase (LDH) were elevated. The patient was treated with 4 cycles of chemotherapy (bleomycin, etoposide, cisplatin) and reached complete remission. Although the finding of a retroperitoneal mass was not accompanied by raised AFP, beta-HCG and LDH, it was considered highly suspicious for recurrent GCT and a retroperitoneal lymphadenectomy was performed. Pathological examination revealed a radically resected teratoma composed of cystically enlarged mature epithelial glands, mature pancreas parenchyma and an acinar cell carcinoma (ACC). There were no radiological abnormalities in the pancreas itself, neither prior to surgery, nor 3 months after the lymphadenectomy. This therefore suggested the development of an ACC in the metastatic teratomatous type II TGCT without a primary tumor in the pancreas.

### Case 2

The second case concerns a male in his fifties in which follow up revealed a mucinous peritoneal tumor, 33 years after orchiectomy with primary surgical resection of thoracic and abdominal metastases of type II TGCT consisting of combined teratoma and seminoma. The current mucinous tumor was actually the fourth malignancy over a period of 33 years. The first tumor was treated with surgery and four rounds of chemotherapy (bleomycine, eoposide and cisplatin) after which complete remission was reached. The second malignancy, 20 years later, was recurrence of his GCT presented as abdominal and thoracic cystic and solid tumors and a highly elevated AFP. This time he was treated with five rounds of chemotherapy (etoposide/cisplatine) and subsequent surgical resection of remaining tumor. Pathological assessment revealed a combined GCT with yolk sac tumor and partly dysplastic teratoma components. Because this tumor was radically resected and AFP normalized after surgery, surveillance policy with intensive follow up was chosen. Four years later a lymphocele was detected. Cytology of cyst fluid did not reveal tumor.

During further follow up he presented with a third malignancy (30 years after his primary TGCT): a solid peritoneal tumor mass on the left side which was surgically resected. Pathological analysis revealed a well differentiated mucinous adenocarcinoma, non-radically resected. This raised the suggestion of an appendicular origin, but there were no signs of a primary carcinoma in the digestive tract after extensive gastro-intestinal examination. Surveillance was continued, and follow up again showed extensive peritoneal tumor mass a year later. This was treated by Hyperthermic Intraperitoneal Chemotherapy (HIPEC) and resection of the peritoneal tumor masses. Pathological examination again revealed a well differentiated mucinous adenocarcinoma. Because of persistent doubts about the possible type II TGCT origin more extensive molecular clonal analyses were performed between the previous teratomatous part of his TGCT and the current carcinoma. Based on these findings palliative chemotherapy (capecitabine/bevacizumab) was started, resulting in stable disease.

## Patients and methods

Appropriate informed consent from the patients was obtained by the treating oncologist. The data of two patients with a history of type II TGCT and a metastatic (retro) peritoneal carcinoma years later was collected from the institutional databases, Cancer Center Amsterdam, Amsterdam UMC and University Medical Centre Utrecht, between 2010 and 2019.

### Immunohistochemistry

For case 1, immunohistochemical stainings using antibodies against Trypsin (DAKO A0012, 1:10000, ARS pH 9.0), Chymotrypsin (DAKO A0022, 1:10000, ARS pH 9.0), Lipase (US Biological L2496.05, 1:200) and BCL10 (Santa Cruz B0315, 1:400) were performed as previously described [16]. Briefly, 4 μm sections were deparaffinized and blocked for endogenous peroxidase activity. After antigen retrieval and blocking of the nonspecific binding sites, the primary antibodies were incubated for 1 h. Antibody binding was visualized using the Brightvision+poly-HRP detection system with DAB as chromogen. For case 2, immunohistochemical stainings for CK7 (Biogenex, United States, MU255-UC, clone OV-TL 12/30, Ventana 1:6400), CK20 (DAKO, United States; M7019, clone Ks20,8, Ventana 1:200), CDX2 (Immunologic, ILM2353-C1, clone EPR2764Y, Ventana 1:200) and SALL4 (Cellmarque, V0000157, clone 6E3) were performed on FFPE slides in Ventana Benchmark Ultra, according to diagnostic procedure protocols used at the pathology department of the University Medical Center in Utrecht, the Netherlands. Stainings were evaluated by 2 independent pathologists.

### Electron microscopy

Small representative pieces of tumor tissue were collected from paraffin embedded formalin fixed tissue. After deparaffination and rehydration, overnight fixation in Karnovsky’s fixative was carried out, followed by postfixation in 1% OsO4 for 1 h at RT. Tissue was dehydrated and embedded in epon, following routine procedures. Ultrathin sections were stained with uranyl acetate and lead citrate and photographed using a transmission electron microscope (Technai12, FEI, Eindhoven, The Netherlands) [17].

### Fish

The presence of 12p was studied by fluorescence in situ hybridization (FISH) on 4 μm sections from buffered, formalin-fixed, paraffin-embedded tissue using the pre-treatment as previously described [18]. Hybridization of the probes and subsequent washes were performed according to the protocol of the manufacturer (Vysis, Abbott Molecular, Hoofddorp, The Netherlands). We used specific probes for the chromosome 12 centromeric (CEP 12 spectrum orange) and telomeric (ETV6 Dual Color Break Apart Rearrangement Probe at the 12p13) region for ploidy determination, whereas probes specifically for chromosome 1 centromere (CEP 1 spectrum orange) and chromosome 6 centromere (CEP 6-spectrum green) served as controls. Two independent observers scored 100 tumor nuclei per section for each probe. FISH and counting was done on the section slides of the original teratoma, the ACC in the metastasis, and a primary pancreatic ACC that was recently reported, as a control [19]. Non parametric testing (two-sample Kolmogorov-Smirnov test) was used to statistically evaluate FISH results for telomeric overrepresentation. In addition, dual color FISH for chromosome 12 centromeric (Chromosome 12 Alpha Satellite Probe (Cytocell LPE012G)) and 12pter Subtelomere (Specific Probe (Cytocell LPT12PR)), was repeated in dual color following the protocol described above to assess the presence of isochromosome 12p. One blinded observer scored at least 150 nuclei per section.

### Whole genome methylation array

DNA isolation was performed as described previously [20]. After bisulfite conversion Illumina’s Infinium MethylationEPIC Kit (850 K array) was used to detect methylation. Copy number variations were determined using the ‘conumee’ package in R as previously described [21].

### SNP array analysis

SNP array copy number profiling and analysis of regions of homozygosity were performed according to standard procedures using the CytoSNP-850 K BeadChip (Illumina, San Diego, CA). Subsequently, visualizations of SNP array results and data analysis were performed using NxClinical software (BioDiscovery, Los Angeles, CA), whereby Human genome build February 2009 GRCh37/hg19 was used.

### Next generation sequencing (NGS)

NGS was performed according to standard procedures using the Ion AmpliSeq™ Cancer Hotspot Panel v2Plus, as described previously [22, 23]. In brief, DNA was isolated using the Cobas method (Roche). DNA concentration was determined using Qubit Fluorometer (Life Technologies). A total of 20 ng of input DNA was used in a final volume of 12 μl. PCR was performed in 20 cycles. Samples were barcoded using IonXpress Barcode Adapters (Life Technologies) to allow for discrimination between samples within a NGS run. The DNA concentration of the samples within one sequencing run were normalized using the Qubit 2.0 fluorometer (ThermoFisher Scientific) or the Ion Library Equalizer kit. The Ion AmpliSeq Library Kit 2.0 (Life Technologies) was used for library preparation. Sequencing was performed using the Ion PGMTM Sequencing 200 kit v2 using the Ion 316TM or 318TM chip (Life Technologies). Sequencing results of the Ion Torrent PGM run were presented via the Torrent Browser, and reads generated were aligned using the Torrent Mapping Alignment Program. Data was analyzed using Coverage Analysis plugin version 3.6 (Life Technologies) and the Torrent Variant Caller plugin version 3.6.

## Results

### Case 1

**Fig. 1:**
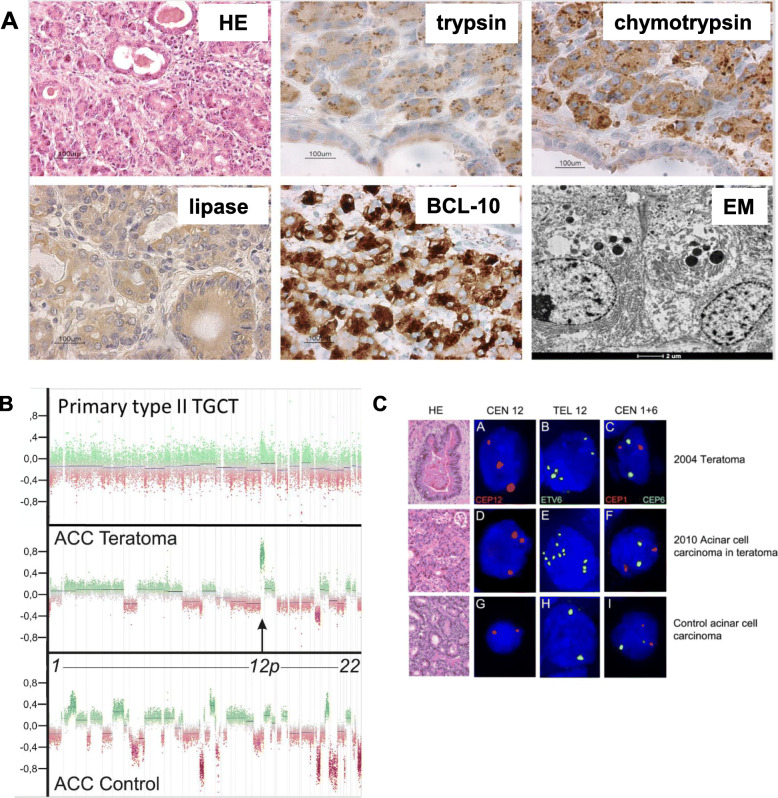
– case 1 **a**. Immunohistochemical confirmation of acinar cell component. **a** HE acinar differentiating within metastatic teratomatous type II TGCT, immunolabeling with antibodies against trypsin, chymotrypsin, lipase and BCL10; electron microscopy of zymogen granules. **b**. Molecular genetic analysis of the ACC within the teratomatous type II TGCT and a control ACC. Arrow pointing to chromosome 12p showing probable amplification in the primary TGCT, and evident amplification in the teratoma ACC compared to control ACC. **c**. Cytogenetic analysis of the primary teratomatous type II TGCT and the ACC. FISH results for centromere (CEN12, A, D, G) and telomere (TEL12, B, E, H) regions on chromosome 12 and control probes (CEN1 and CEN6, C, F, I)

Morphologically a diagnosis of ACC within the retroperitoneal teratomatous type II GCT was suspected (Fig. 1a). Immunohistochemistry for trypsin, chymotrypsin, lipase and BCL10 (Fig. 1a) was positive, consistent with acinar differentiation. Furthermore, electron microscopy (EM) showed the presence of the characteristic zymogen granules (Fig. 1a). Therefore, immune-morphological and EM data were consistent with an ACC, apparently arising in a metastatic cystic teratomatous type II GCT.

Copy number variation (CNV) analysis based on EPIC methylation data confirmed a gain of 12p in the acinar cell component of the metastatic malignancy but not in the control ACC (Fig. 1b). In addition to 12p gain in the metastatic teratoma multiple possible CNVs were observed including loss of 1p36 and 16q (Fig. 1b). The control primary ACC showed multiple losses and gains, different from the ACC component in the teratomatous type II GCT (Fig. 1b). Although the quality of the assay was low, the methylation data of the primary type II TGCT also suggested 12p gain Fig. 1b). There was no evident suspicion of any other CNVs, suggesting that loss of 1p36 and 16q could be signs of progression. However, the spread in the CNV plot of the primary teratoma is too large to exclude CNVs with certainty.

FISH identified overrepresentation of 12p in the primary type II TGCT and the ACC component of the metastasis. A control primary pancreatic ACC was also analyzed and showed no CNVs of 12p (Fig. 1c). The median number of copies per nucleus was 3 for the centromere probe (CEN12) and > 4 for the 12p telomere (ETV6) probe (*p* < 0,01) in both the primary type II TGCT and the ACC. Control probes for the chromosome 1 and 6 centromere revealed no chromosomal abnormalities. Repeated FISH with dual color probes was performed in order to evaluate presence of isochromosome 12p, which was not convincingly shown.

### Case 2

**Fig. 2:**
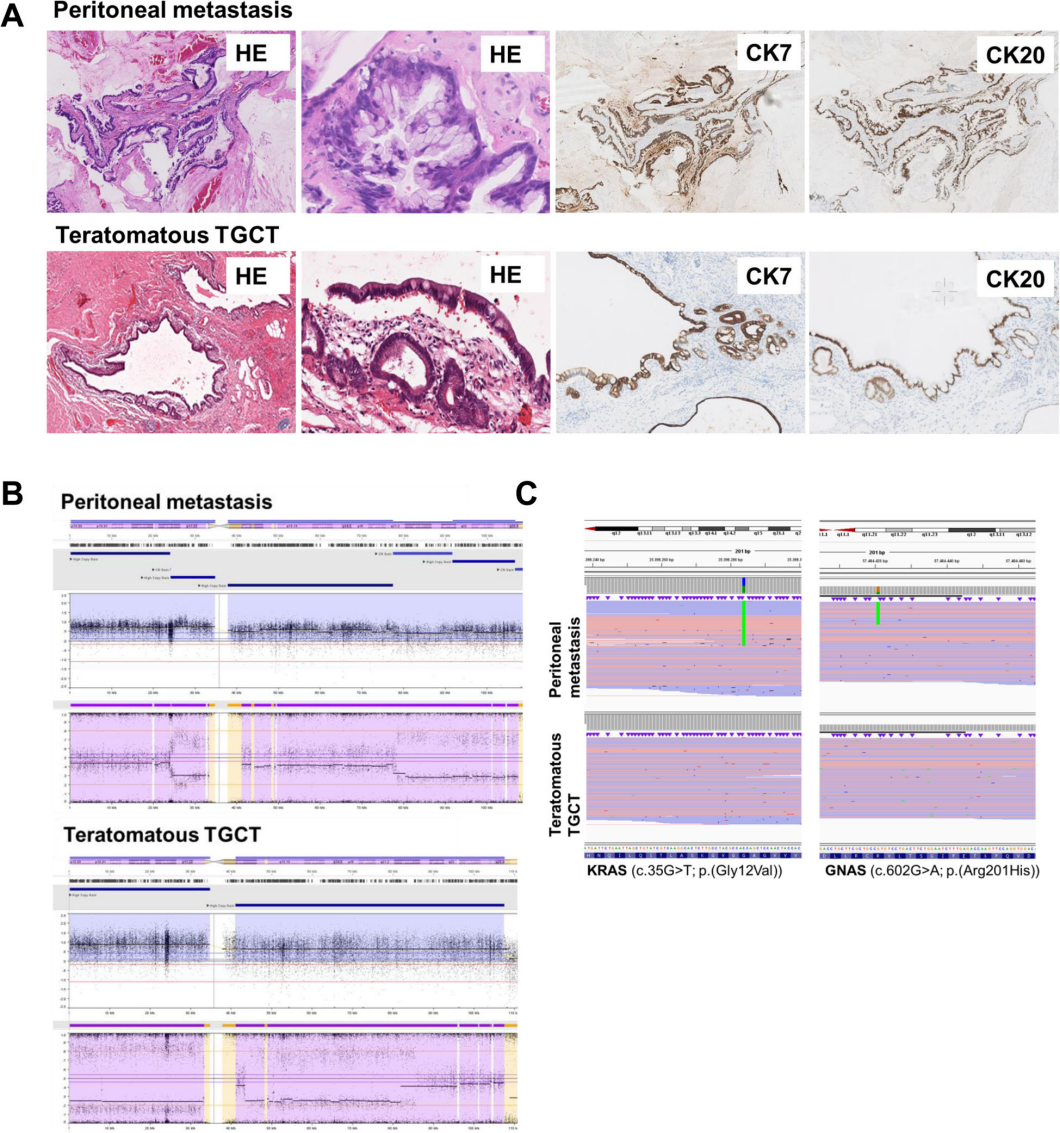
– case 2 **a**. (Immune) histochemical similarities of the peritoneal metastasis and the dysplastic intestinal component of the teratomatous type II TGCT. HE stained slides at different magnifications and immunolabeling with antibodies against CK7 and CK20. **b**. Molecular genetic analysis of the peritoneal metastasis and the dysplastic intestinal component of the teratomatous type II TGCT by SNP array. Chromosome 12 is shown in detail which shows a different pattern in gains and losses of both tumors: high gains of the entire 12p and a part of 12q and CN-LOH of 12q in the teratomatous type II TGCT and multiple partial high gains of chromosome 12p and a partial high gain of 12q and multiple gains of chromosome 12q in the metastasic adenocarcinoma. **c**. Molecular genetic analysis of the peritoneal metastasis and the dysplastic intestinal component of the teratomatous type II TGCT by NGS. The peritoneal metastasis showed a mutation in KRAS and GNAS while the primary teratomatous type II TGCT showed no mutations in the genes investigated in this panel

The current peritoneal metastasis showed intestinal differentiation with dysplasia, morphologically resembling the metastasized teratomatous type II GCT resected 20 years after his primary tumor (Fig. 2a). Therefore, additional immunohistochemical staining was performed which showed positivity for cytokeratin 7, cytokeratin 20 and Caudal Type Homeobox 2 (CDX2) in both the current peritoneal metastasis as well as the previous teratomatous type II GCT metastasis (Fig. 2a and supplementary figure 1). Glypican 3, Octamer binding transcription factor (OCT3/4), placental alkaline phosphatase (PLAP) and AFP were negative (data not shown). Sal-like protein 4 (SALL4) did show some expression in a minority of CDX2-positive glands (supplementary figure 1). Based on the immune-morphological similarities, expression of some SALL4 and the fact that immunereactivity for germ cell markers is often lost in a teratoma with somatic type malignancy [14], the peritoneal metastasis could very well be related to the previous teratomatous TGCT.

To further substantiate this suggestion, a SNP array was performed to evaluate probable additional molecular similarities between the dysplastic intestinal component of the teratoma and the current peritoneal mucinous metastasis, in particular 12p aberrations. To our surprise, CNV analysis based on SNP array data did not show a clonal relationship. SNP array showed a high gain of the entire 12p in the intestinal component of the metastatic teratomatous GCT20 years after his primary TGCT, while only a partial gain of 12p was present in the current peritoneal mucinous tumor (Fig. 2b). Furthermore, the teratomatous GCT showed numerous chromosomal imbalances which were structurally different from the copy number variations of the peritoneal metastasis. For example copy number neutral-loss of heterozygosity (CN-LOH) of chromosome 4 in the teratoma and CN-LOH of chromosome 4p, partial gain of 4q and a partial loss of 4q in the peritoneal metastasis (see supplementary data for detailed information).

To further confirm that these tumors were not clonally related and to investigate the possible primary origin of the peritoneal tumor, NGS was performed using Ion AmpliSeqTM Cancer Hotspot Panel v2Plus. This showed mutations in KRAS exon 2 (c.35G > T; p.(Gly12Val), VAF 45%]) and GNAS (c.602G > A; p.(Arg201His), VAF 33%]) in the current peritoneal tumor, while no mutations were found in the metastatic teratomatous TGCT 20 years after his primary TGCT (Fig. 2c). Although this could indicate tumor progression of the peritoneal metastasis, the presence of both KRAS and *GNAS* mutations in particular point towards peritoneal metastasis of a primary mucinous tumor of the appendix or pancreas [24–26]. This also fits with the peritoneal localization of the metastasis.

## Discussion

The cases described in this article underscore the important and decisive role of additional molecular testing in patients with a differential diagnosis between somatic metastasis of type II TGCT and a second primary malignancy, with significant impact on diagnosis, treatment and prognosis.

Both cases share a similar dilemma: a metastatic epithelial malignancy in a patient with a history of teratomatous type II TGCT. As both metastases presented more than 2 years after the completion of first-line chemotherapy for advanced TGCT, it would be considered a late recurrence in which GCTs with somatic differentiation is frequently present [12–14]. .This poses the challenging differential diagnosis between somatic transformation of teratoma, or a new non-germ cell malignancy.

Based on morphological and immunohistochemical analysis alone, the two most commonly used diagnostic tools in routine pathology, both carcinomas appeared to be metastasized somatic transformation of teratoma. Whether the epithelial malignancy is a metastasis of the TGCT or from another yet unidentified primary neoplasm makes a great difference with regard to choice of treatment and prognosis. Therefore, it is of great importance to be able to state the origin of the current metastatic carcinoma with certainty. Molecular techniques are relatively expensive and time consuming which is why many ISUP-associated pathologists confine to histological and immunohistochemical assessment, as surveyed in a recent manuscript related to an international survey regarding the application of immunohistochemistry and molecular pathology on the diagnosis of testicular germ cell tumors [8].

To our knowledge, this is the first report of an ACC arising in a metastasized teratoma verified by molecular analysis in addition to immunohistochemistry and electron microscopy. The occurrence of somatic-type malignancies from type II TGCTs is uncommon, but has been reported in approximately 3–6% of metastatic germ cell tumors [2, 27]. The most frequent histological subtypes are rhabdomyosarcoma, adenocarcinoma and primitive neuroectodermal tumors. In rare cases adenocarcinoma with acinar differentiation has been reported [13, 15]. In the first case, FISH and whole genome methylation array results were consistent with a germ cell origin of the ACC which saved both the patient and doctors the efforts of searching for a yet unidentified primary pancreatic neoplasm.

Regarding the second case, based on the immune-morphological profile the peritoneal metastasis of a mucinous adenocarcinoma was considered to originate from the previous metastasized teratomatous type II TGCT. Such morphological and immunohistochemical matches have long been the standard in proving a clonal relationship, which has also been reported in literature of a similar case [28]. However, SNP array showed many subtle but clear differences in CNVs between both tumors, revealing that the two malignancies could not be clonally related. Furthermore, the combination of KRAS and *GNAS* mutation discovered in the well differentiated peritoneal mucinous adenocarcinoma metastasis indicated that a primary low-grade appendicular neoplasm (LAMN) was the most likely primary origin. Interestingly, a KRAS V12G mutation in particular was observed most frequently in genome-wide mutational analysis of low grade mucinous carcinomatosis peritonei of appendiceal origin [26]. Of note, an endoscopy a few months before the metastasis did not show any abnormalities in the gastro-intestinal tract. Above all, there was a normal base of the appendix. It is unclear from the status of the patient whether or not his appendix was removed previously, underlining the importance of pathological assessment of resected specimens, even without any clinical suspicion of significant pathology.

In case 2 we were able to perform SALL4 staining on the peritoneal metastasis, which did show focal expression. This expression in particular could be misleading as it is regarded a sensitive and specific marker for metastatic germ cell tumors [29]. However, SALL4 expression has been detected in non-GCT related somatic type malignancies as well [8, 30]. Therefore, even though this markers is most robustly present in teratoma with somatic type malignancy compared to PLAP, OCT3/4 and a-FP, it’s expression does not necessarily point towards TGCT origin [8].

Therefore, without the molecular analyses, there would be an ongoing ambiguity about the precise origin of the current retroperitoneal metastasis of case 1, with subsequent uncertainty and intensive, unnecessary, follow up for the patient. Case 2 would have been regarded as a somatic-type malignancy developing from a TGCT in a metastatic site, with obvious mismanagement regarding choice of therapy. Therefore, this report underscores the important, or even obligatory, contribution of molecular analyses in nowadays diagnostic pathology [8, 31].

## Conclusion

Taken together, the definitive diagnosis and patient management in these cases was determined by molecular diagnostics and enabled a personalized treatment and follow up for each patient. This is in line with recent recommendations from the ISUP which state that molecular tests like FISH, SNP array, gene sequencing and methylation array can be used to confirm origin of (metastatic) type II TGCT, as well as for ovarian and extragonadal GCTs.

## Supplementary information

**Additional file 1: Supplementary Materials.** The following are available online at www.mdpi.com/xxx/s1, **Figure S1.** SNP array analysis of the teratomatous TGCT and the peritoneal metastasis of case 2.

## Data Availability

The dataset supporting the conclusions of this article is included within the article (and its additional file).

## Abbreviations

ACC: Acinar cell carcinoma
AFP: Alpha-fetoprotein
CDX2: Caudal Type Homeobox 2
CN-LOH: Copy number neutral-loss of heterozygosity
CNV: Copy number variation
FISH: Fluorescence in situ hybridization
Beta-HCG: Human chorionic gonadotropin-beta
ISUP: International Society of Urological Pathology
LAMN: Low-grade appendicular neoplasm
LDH: Lactate dehydrogenase
NGS: Next generation sequencing
OCT3/4: Octamer binding transcription factor
PLAP: Placental alkaline phosphatase
SALL4: Sal-like protein 4
SNP-array: Single nucleotide polymorphism (SNP)-array
TGCT: Testicular germ cell tumor
