# Supplementary material for: The decisive role of molecular pathology in presumed somatic metastases of type II testicular germ cell tumors: report of 2 cases
Source: Diagn Pathol. 2020 Jul 25;15:99. doi: 10.1186/s13000-020-01011-0 (PMC7382836; doi:10.1186/s13000-020-01011-0)

**Supplementary data**

**
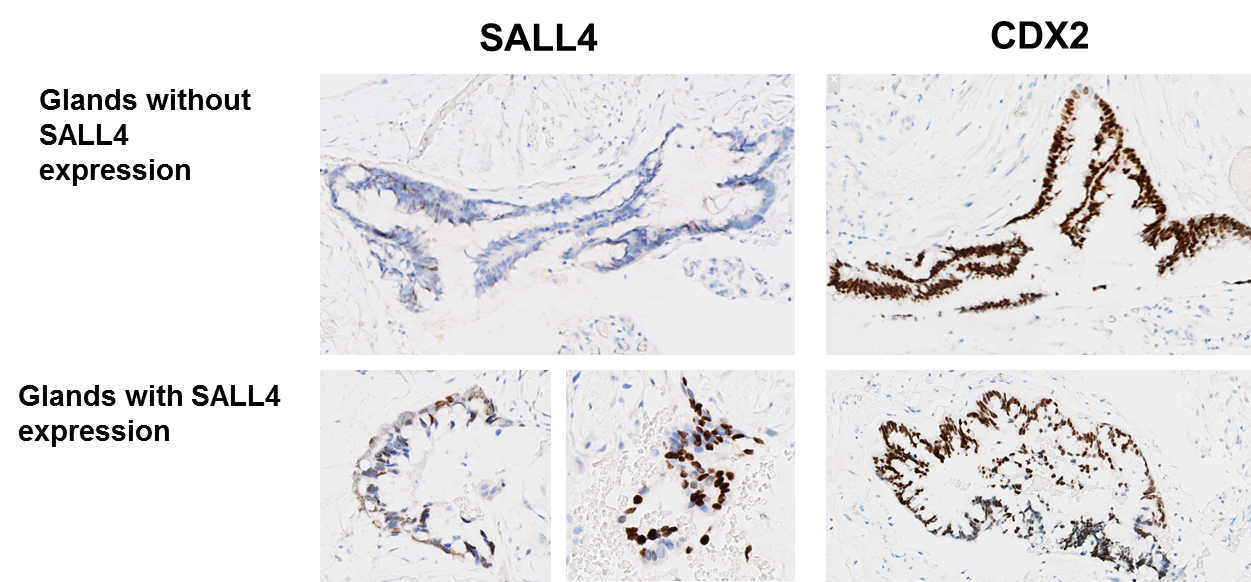
**

**Figure 1. Immunohistochemical expression of SALL4 and CDX2 in the peritoneal metastasis.**

**SNP array analysis**

*Summary of findings of the dysplastic epithelial component of the teratomatous TGCT of 2007*

There appeared to be a gain of chromosome 3p and a partial gain and partial high gain of chromosome 3q; a CN-LOH of chromosome, 4; a high gain of chromosome 5p and CN-LOH of chromosome 5q; a gain of chromosome 8p; high gains of chromosome 12p and a part of 12q and CN-LOH of 12q.

*Summary of findings of the peritoneal metastasis*

There appeared to be tetrasomy of chromosome 3; CN-LOH of chromosome 4p, partial gain of 4q and a partial loss of 4q; a gain of chromosome 5p and a partial gain of chromosome 5q; a partial CN-LOH of 8p, partial gain of 8p and a gain/high gain of 8q; multiple partial high gains of chromosome 12p and a partial high gain of 12q and multiple gains of chromosome 12q.

**CNV profile of the SNP array of teratomatous TGCT of 2007**
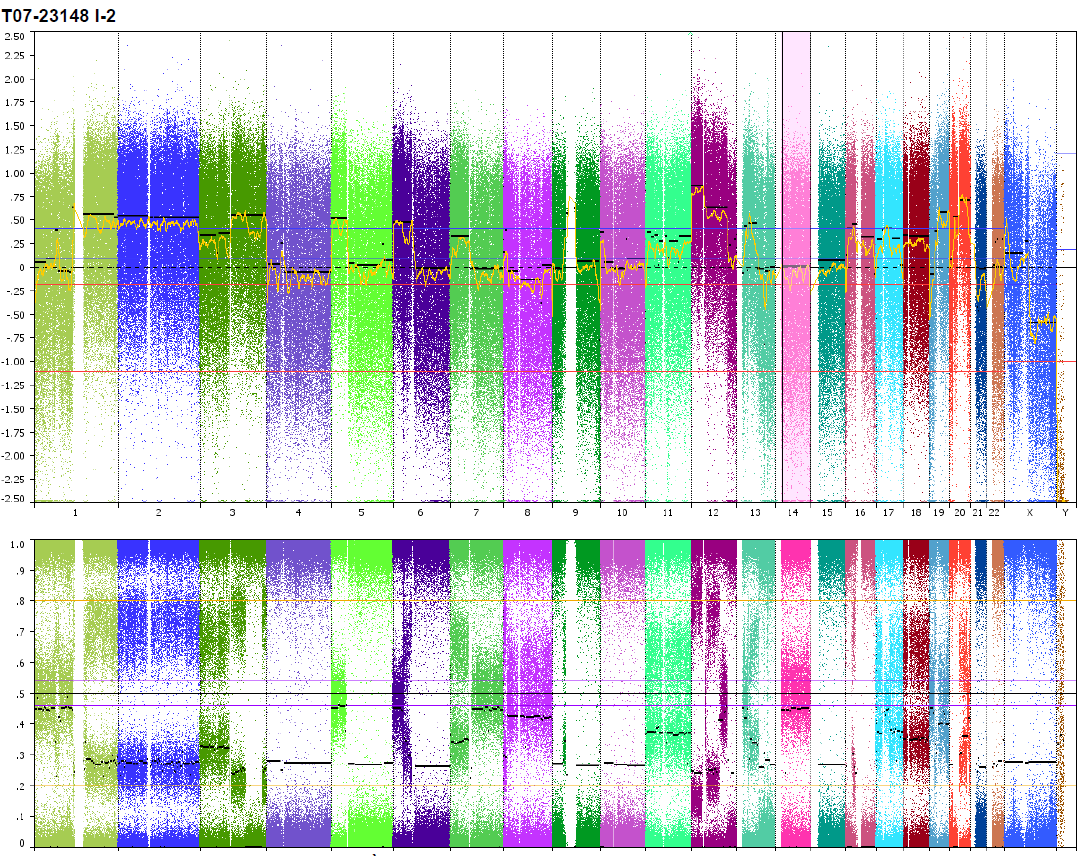


**CNV profile of the SNP array of the peritoneal metastasis**


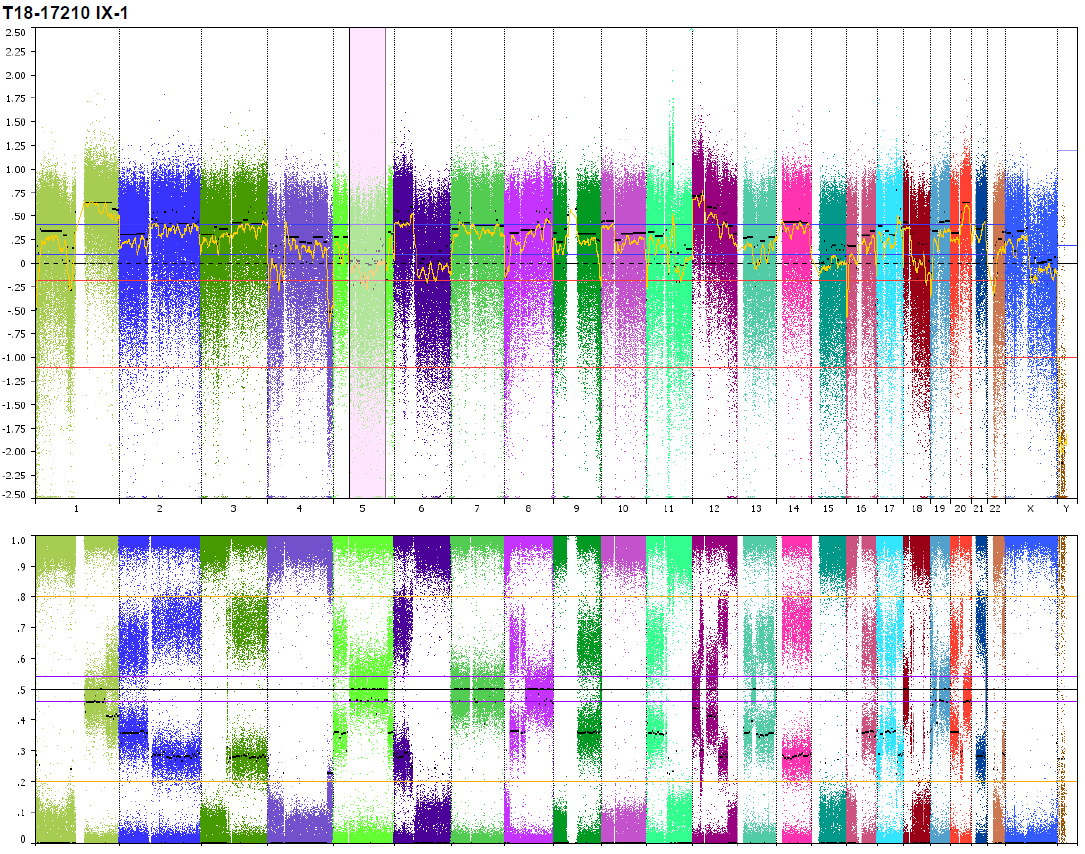

Supplement: Supplementary file 1 — Additional file 1: Supplementary Materials. The following are available online at www.mdpi.com/xxx/s1, Figure S1. SNP array analysis of the teratomatous TGCT and the peritoneal metastasis of case 2. [file 13000_2020_1011_MOESM1_ESM.docx]
